# Economic Cost of Current and Alternative Models of Multidisciplinary Care of Juvenile‐Onset Huntington's Disease

**DOI:** 10.1002/mdc3.70433

**Published:** 2025-11-11

**Authors:** Tracey A. Young, Penny A. Curtis, Jill Thompson, Aileen K. Ho, Helen Santini, Oliver W. Quarrell

**Affiliations:** ^1^ Health Economics and Decision Science, Sheffield Centre for Health and Related Research, School of Medicine and Population Health University of Sheffield Sheffield UK; ^2^ School of Allied Health Professions, Nursing and Midwifery University of Sheffield Sheffield UK; ^3^ School of Psychology and Clinical Language Sciences University of Reading Reading UK; ^4^ Advisory Service, Huntington's Disease Association, Liverpool Science Park IC1 Liverpool UK; ^5^ Sheffield Children's Hospital, Western Bank Sheffield UK

**Keywords:** Huntington's disease, Juvenile‐onset, economic cost, multidisciplinary team, virtual clinics

## Abstract

**Background:**

Multidisciplinary care has been advocated for Juvenile‐onset Huntington's Disease but there has been no detailed analysis of this.

**Objectives:**

To evaluate the current economic costs of providing health care for patients with Juvenile‐onset Huntington's disease (JoHD) and to model the effects and economic costs of providing a multidisciplinary team (MDT) approach.

**Methods:**

Patients were recruited through the patients’ organization, the Huntington's Disease Association, and specialist Huntington's disease clinics. Thirty‐three adapted Client Service Receipt Inventories were completed following either a telephone or in‐person interview. The effects of delivering two models of providing a systematic MDT approach, traditional in‐person MDT and digital MDT were assessed.

**Results:**

The overall mean JoHD cost to the NHS per year (2020) was £126,966 which varied according to disease severity, costing just under £11,000 in mild disease, £132,419 in moderate disease, and £221,797 in advanced JoHD. The proportion of indirect costs to families and carers was 64% for mild disease and 17% for advanced disease. Assuming a 50% reduction in health care professionals seen, the alternative models saw reduction costs of £2490 and £2730, respectively.

**Conclusion:**

We document the high cost of care in JoHD, particularly as the disease progresses towards the later stages. We present estimates from modeling the effects of providing a systematic MDT approach to the care of patients and families with JoHD. These data have clear implications in the consideration of future JoHD models of care in an increasingly digital age and can be developed further as disease modifying treatments become available.

Huntington's disease (HD) is a progressive neurodegenerative disorder inherited as an autosomal dominant condition characterized by a movement disorder, cognitive impairment and affective disturbance,^1^, ^2^, ^3^ profoundly impacting health‐related quality of life.^4^ It is caused by an expansion of a CAG repeated sequence in the first exon of the *HTT* gene.^5^ There is currently no treatment to alter the natural history of the condition, but clinical trials of potential treatments have been, and continue to be, undertaken.^6^

While onset can occur at almost any age, the average onset age is in the fifth decade of life.^2^ There is an inverse correlation between the length of the expanded CAG repeat and the average age of onset. Traditionally those with onset of ≤20 years have a very high CAG repeat length and are described as having juvenile‐onset HD (JoHD), and in economically developed countries this represents approximately 5% of cases.^6^

There is a limited evidence base for the management of patients with JoHD and still less information on the costs of managing young people with JoHD. There have been calls from an expert group for a multidisciplinary team (MDT) to form around the young person affected by JoHD: these calls include descriptions of the MDT but have not been evaluated.^7^, ^8^ Currently, MDTs form on an ad hoc basis because the condition is rare, and individual cases are widely dispersed. There is considerable heterogeneity of methods, time and geography in HD prevalence studies.^9^ The prevalence of HD in the UK studies varies between 5.93 and 14.6 × 10^−5^ ^10^, ^11^ implying that there are 4000–10,000 cases and suggests JoHD represents between 200 and 500 cases. At present the costs of JoHD within a UK setting are unknown. A recent systematic review of the economic evaluation of HD did not focus on JoHD nor did a further study that included UK patients.^12^, ^13^ Therefore, making it important to first understand the current costs of JoHD before assessing possible alternative models of care. Based on our experience, two scenarios were considered for modeling purposes: one was a traditional face‐to‐face MDT with professionals and families traveling to a specified site and liaising with the local team, Figure 1; the other involved professionals staying in their own location and using information and computing technology to communicate with each other, families and liaising with the local team, Figure 2.

**Figure 1:**
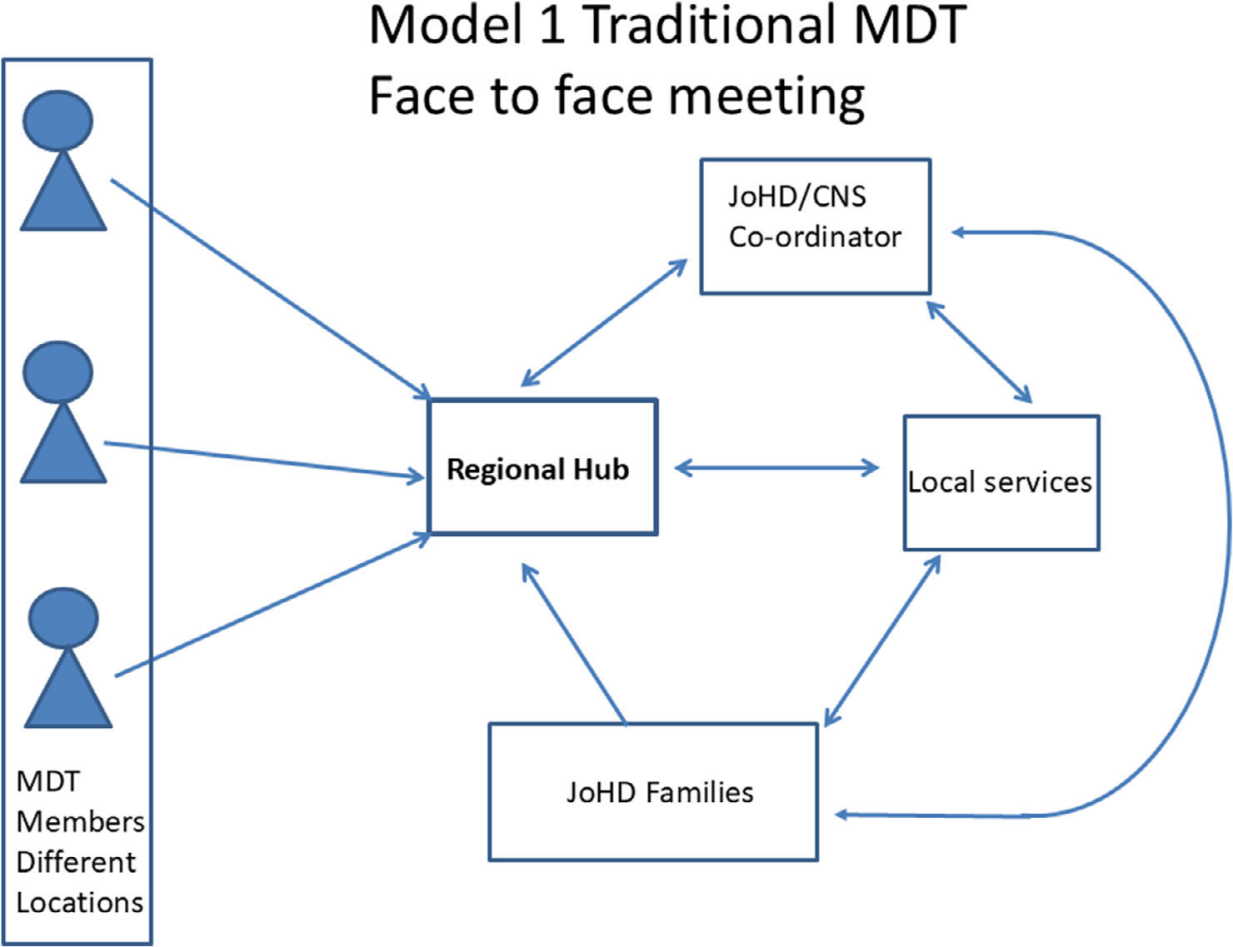
Model 1 Traditional MDT with face‐to‐face meeting. Members of the specialist JoHD MDT and families and representatives of local services travel to specific sites. The MDT work and liaison with families and local service providers is facilitated by the JoHD coordinator, a clinical nurse specialist (CNS) or equivalent professional.

**Figure 2:**
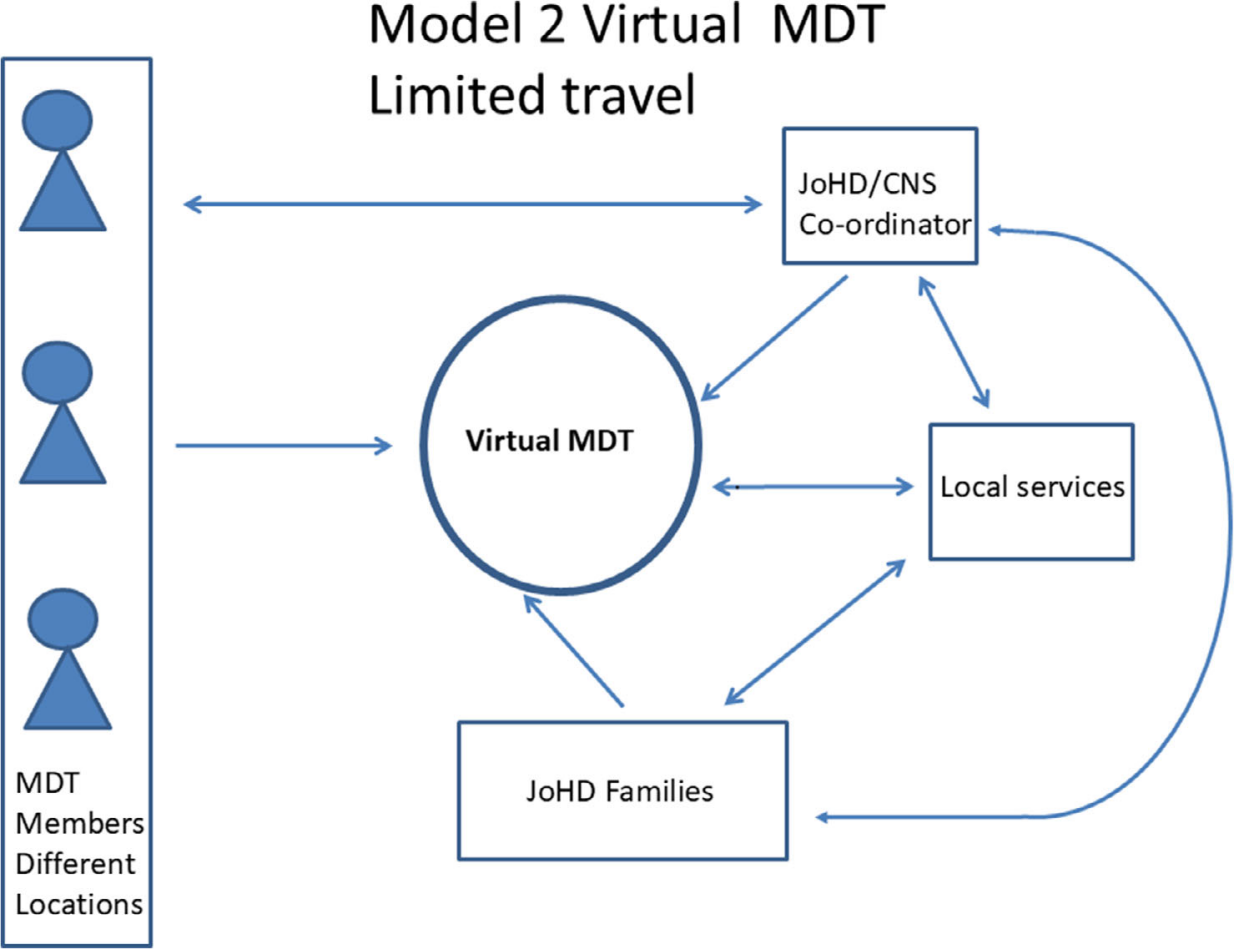
Model 2 A Virtual MDT with limited travel. The JoHD coordinator (CNS or equivalent professional) liaises directly with the families and members of the specialist MDT who are different locations but can meet with families and members of the local services virtually.

In this paper we document the economic cost of current care in JoHD over a one‐year timeframe and estimate the financial costs of two proposed models of care: this will aid future planning as and when disease modifying treatments become available.

## Methods

### Patient Recruitment

To estimate the costs of JoHD we recruited JoHD families throughout England and Wales. Given that JoHD is rare, patient identification for this study was therefore mainly via the patients’ organization, the Huntington's Disease Association (HDA). Additional participants were recruited through multiple HD specialist clinics. Study information was also circulated via a social media account and the HDA newsletter, website and social media feeds. Ethical approval for the study was given by Yorkshire and Humber Leeds West Research Ethics Committee 14/YH/1032.

### Sample and Data Collection

Owing to the wide dispersal of both the JoHD patients/families throughout England and Wales and the resource use across multiple services it was not possible to obtain resource use information from routine data sources. Therefore, a resource use questionnaire was adapted from the client service receipt inventory (CSRI).^14^ The adaptation was based on pilot interviews with families and developed by the project team aided by two family members who acted as advisors. The questionnaire asked about resource use over the past 12 months related to: health and social care services accessed, professionals seen, current JoHD medications, medical investigations, equipment adaptations, support with daily activities and changes to employment or financial circumstances. Information was also sought about whether the resources were related to JoHD and whether they were accessed by the NHS (pediatric or adult) or local authority, privately (self or family), from a charity or voluntary organization. The questionnaire also asked about resources borne by the person with JoHD, or their family, were also collected. The questionnaire was administered via interview either face‐to‐face or over the phone and was piloted prior to data collection. Between October 2015 and February 2017, the questionnaire was sent in advance to people with JoHD and was either completed by the interviewer with them and/or with their carer. A copy of the questionnaire can be found in Supporting Information S1.

### Estimates of Care Costs

A unit cost for each item of resource use indicated in the resource use questionnaire is needed to estimate the care costs for JoHD. Table 1 summarizes the types and sources of unit costs, with a detailed list of the unit cost source and any assumptions made for each item of resource use available in Supporting Information S2. Unit costs were obtained for resource use items identified as having been provided by the NHS, social care or local authority, criminal justice system and alternative therapies. Travel to and from appointments was reported to be by car, taxi or bus and was costed accordingly. Resources borne by the person with JoHD, or their family, included alternative therapies, equipment, adaptations, carer time (formal and informal) and changes to employment or financial circumstances.

**TABLE 1:** Summary of the sources of the unit cost of care

General source	Specific unit cost source	Reference
NHS and Social care	NHS national cost collection	15
	Personal Social Service Research Unit (PRSSU)	16
	British National Formulary (BNF)	17
	Royal College of Nursing Agenda for change for staff costs 2019/20^a^	18
	NHS Employers: pay and Conditions Circular (M&D) 2020^a^	19
Prison Costs and Police time	UK Government; Heslin et al	20, 21
Alternative therapies	Reflexology and acupuncture websites	22, 23
Transport to and from appointments by bus car or taxi	UK Government website for expenses and benefits of business travel mileage	24
	Taxi calculator including cost per mile plus a standard pick‐up cost	25
	Bus fares were assumed to be £2 per journey as applied under the government fare cap 2020	26
Average cost of a stair lift	Which 2020	27
Carer time provided by family and changes in financial circumstances	Average national wage obtained from the annual survey of hours and earnings	19

^a^ Salary taken from mid‐point of salary scale.

Unit costs were applied to resource use and summed together to obtain an overall cost. Costs are presented as means with 95% confidence intervals obtained using bootstrapping, using 5000 bootstrap replicates. Costs are presented for the year 2019–20. Any unit costs sources prior to 2020 were inflated to 2020 prices using inflation indices from PSSRU 2020.^16^

A total cost per person with JoHD was estimated over a one‐year time frame. Costs are not discounted due to the annual time frame. The primary analysis presents costs borne by the NHS and social care services. Two further analyses were carried out: (i) examining costs to the wider payer provider (including NHS and social care costs, police and criminal justice system and education costs), (ii) costs from a broader perspective (including costs to the person with JoHD and their family, NHS and social care costs, police and criminal justice system and education costs).

### Sub‐Group Analysis: Assessment of Disease Severity

Costs of JoHD were examined by JoHD disease severity. Assessment of the severity of JoHD (mild, moderate or advanced) was made for 31 cases (two cases could not be contacted for severity assessment). Although the Shoulson and Fahn Total Functional Capacity Scale (TFC)^28^ is frequently used for this purpose in HD, this was considered inappropriate as it includes questions that have limited relevance for the younger JoHD age‐range. An empiric severity assessment was therefore undertaken by two of the authors (OQ and HS), by asking about onset of symptoms, age of diagnosis and whether individuals’ abilities to undertake activities of daily living were mildly, moderately or severely impaired. From the answers a clinical judgment of the disease stage was made.

### Estimate of the Costs of Alternative Multidisciplinary Models of Care with Specialist JoHD Experience or Support

Two alternative models of care were hypothesized following discussion with the project team and the two family member advisors using a previously published list of professionals involved in the care of JoHD patients as a starting point.^7^

Traditional face‐to‐face model (Model 1, Fig. 1) consisted of a national co‐ordinator and three regional hubs with expert teams, where each regional hub would convene twice a year to discuss care with families, and up to six families could be seen in any one day. Experts and families would travel to the regional hub for the consultations and the aim would be for each family to be seen once a year. Families could also be seen by their local team at the same meeting as well as on other occasions.

In Model 2 (Figure 2) rather than experts and families traveling to local hubs, a virtual network would be set up. Families would still be seen once a year, though they would only need to travel to their local center for a virtual meeting with experts rather than to a regional hub.

For both models it was envisaged employing two part time workers 0.6 full time equivalent for the national clinical coordinator role, a role analogous to a clinical nurse specialist within the NHS (Annual salary £38,765^18^), or an equivalent clinical professional. The national clinical co‐ordinator would be required to liaise with the local teams, people with JoHD and their families and with local experts outside of the team meetings. It was assumed that one day of phone calls per JoHD patient per year would be required to enable the coordinator to communicate with everyone.^29^ Further, it was assumed that room hire for the hubs would be at an additional cost.^1^

The expert panel would be compromised of:

1. Neurologist or pediatric neurologist.
2. Speech and language therapist.
3. Depending on the requirements of the child or young person, as assessed by the national coordinator, an occupational therapist or physiotherapist.
4. Palliative care consultant.
5. Adult or pediatric psychiatrist (Assume mid‐point of BMA consultancy salary.
6. Social worker.

The local team, as a minimum, would consist of:

1. A consultant seen three times a year.
2. Depending on the needs of the child or young person an occupational therapist or physiotherapist seen three times a year.
3. Social worker seen three times a year.

For Model 2 it was assumed that ½ days’ worth of IT assistance would be needed per month of a band 5 IT person (Assume mid‐point band 5 agenda for change: £26,220 per annum or £13.45 per hour^15^). Further, the virtual model would require a platform to host the consultations.^30^ Supporting Information S3 provides a breakdown of costs for each model.

The estimated cost to the NHS of a model of care applied nationally was estimated, assuming a total of 36 people with JoHD would be seen per year. This assumption is based on three patients being assessed in a monthly clinic. It was assumed that the person with JoHD and two family members would attend each consultation, and the families would travel approximately 100 miles each way to consultations under model 1, and 10 miles each way under model 2. Costs borne by the families were included in sensitivity analyses.

In order to estimate the cost savings to the NHS of the national model of care for JoHD, the expected costs per patient per year were compared with costs of current care (care costs). Three scenarios were run:

1. The national system had no impact on current care and people with JoHD continued to access a variety of health care professionals;
2. As a result of the national model, people with JoHD no longer needed to access other health care professionals and the additional NHS cost of other professionals seen was £0;)
3. As a result of the national model people with JoHD halved the number of other health care professionals seen.

Further, an estimate as to whether the reduction of health care professionals seen would save money for the NHS, was estimated, based on a national model of care.

The software package STATA v17 was used for all analysis.^31^

## Results

A total of 33 people with JoHD were interviewed about resource use. At the time of the interview the mean age of the person with JoHD was 23.0 years (SD = 6.13) range from 7 to 38 years, with 17 (51.5%) responders being male. The majority of people with JoHD were single (*N* = 31) with two people reporting that they were in a relationship. Three people with JoHD had children, two of whom had two children; age range of the children was 2–8 years. Of 31 people with JoHD, 10 (32%) were categorized as mild, seven (23%) were moderate and 14 (45%) were advanced. In two cases it was not possible to make a classification.

A companion (family or carer) was present in 27 interviews. The average age of the companion was 54.3 years (SD = 9.40) range from 38 to 73 years.

Table 2 presents the current living arrangements for people with JoHD. Most people with JoHD were living at home with family, with seven people in residential care. Table 2 also presents the current schooling or occupation of people with JoHD. One third of people were in school and a further 27% were in college, seven (21%) were in specialist education or college, 6% were employed (full or part‐time) and 6% were unemployed at the time of the interview.

**TABLE 2:** Current living arrangement of and current or previous education or occupation of JoHD patients

Where person with JOHD currently living	*N*	Percent
At home with family	19	57.6
Permanent residential care	7	21.2
Separate home outside family with support	2	6.25
Prison	1	3.03
At home with foster carers	2	6.25
Separate home outside family no support	1	3.03
Missing	1	3.03
Schooling or occupation		
Current		
Mainstream school	11	33.3
College	9	27.3
Specialist educational establishment	6	18.2
University	2	6.25
Unemployed	2	6.25
Specialist educational establishment (College)	1	3.03
Part‐time employment	1	3.03
Full‐time employed	1	3.03

An Education, Health and Care Assessment had been provided in 13 cases (39.4%) but had not been provided in 19 cases (57.6%) and was unknown for one person. Of those, where it had been or was currently provided, the mean age at provision was 11.7 years (SD = 4.64, range 4 to 20).

### Overall Costs

Over 48% of people with JoHD accessed A&E (Table 3) with over 60% accessing GP, speech and language therapists, neurologists or dieticians (Supporting Information S2). Most participants needed bathing equipment, wheelchairs or the installation of handrails and over 60% needed some form of help with daily activities (Supporting Information S2).

**TABLE 3:** Services accessed

	Number using service (%)	Mean number of visits
A&E	16 (48.5%)	0.94 (1.37) (0 to 6)
Residential/nursing home care	8 (24.2%)	7 permanent residents and 1 stay of 104 days 80.6 days
Respite care	7 (21.2%)	7.55 days (0 to 104)
Day center visits	3 (9.1%)	7.88 days (0 to 156)
In‐patient (acute care)	3 (9.1%)	0.03 days (0 to 1)
In‐patient (mental health)	2 (6.1%)	0.03 days (0 to 1)
Other: prison	1 (3.0%)	

The average cost per year to the NHS for each person with JoHD is £126,966 (95% CI: £79,411 to £188,570) and is higher for those with advanced JoHD (Mean = £221,797 (95% CI: £141,941 to £346,264)) compared with moderate (Mean = £132,419 (95% CI: 37,452 to £226,503)) and mild JoHD (Mean = £10,845 (95% CI: £3794 to £19,647)) (Table 4). Costs to people with JoHD, their families and carers make up 21% of the overall cost of JoHD, it should be noted that for those with mild JoHD families incur 64% of costs, whereas those with moderate and advanced JoHD incur 19% and 17% of costs, respectively, reflecting the increased care costs by the NHS and local authority with disease progression.

**TABLE 4:** Summary of total costs

		No. accessing	Mean (SD)	95% bootstrap CI
NHS/LA costs	Mild	10 (100%)	£10,845 (£12,588)	£3794 to £19,647
	Moderate	7 (100%)	£132,419 (£126,013)	£37,452 to £226,503
	Advanced	14 (100%)	£221,797 (£190,087)	£141,941 to £346,264
	All	33 (100%)	£126,966 (162,820)	£79,411 to £188,570
Costs to family	Mild	9 (90%)	£19,269 (£17,995)	£8272 to £30,758
	Moderate	7 (100%)	£33,118 (£45,657)	£9892 to £74,687
	Advanced	14 (100%)	£48,696 (£55,825)	£22,559 to £77,829
	All	32 (97.0%)	£35,993 (£43,918)	£22,616 to £51,980
All costs	Mild	10 (100%)	£30,182 (£24,825)	£15,280 to £45,788
	Moderate	7 (100%)	£177,736 (£134,776)	£77,887 to £279,841
	Advanced	14 (100%)	£289,363 (£220,351)	£197,929 to £431,370
	All	33 (100%)	£173,575 (£190,673)	£118,350 to £246,574

### Alternative Models of Care

The estimated cost to the NHS of an alternative model of care for JoHD was £1787 per person per year under Model 1, and slightly less, at £1574 per person per year under Model 2. Meeting room costs vary between NHS Trusts, so costs were slightly more if an alternative meeting room cost was used (£1796 Model 1; £1580 Model 2) and if costs to families and their carers were included in estimates (£2099 Model 1 and £1700 Model 2). However, in all cases, Model 2, using virtual clinics, would be less expensive than Model 1 per patient per year.

The current annual cost per patient to the NHS and Local Authorities for JoHD was estimated to be £126,966. If a new model of care were introduced, it could impact on the number of health care professionals seen. Table 5 presents potential cost savings to the NHS under three alternative scenarios. If the number of health professionals seen by the NHS were reduced, then savings to the NHS may be £6767 (eliminating other health professionals seen) or £2490 (reduced by 50%) under Model 1 and £6980 (eliminating other health professionals seen) or £2703 (reduced by 50%) under Model 2. Furthermore, for Model 1 to be cost saving to the NHS, it would require health professionals’ costs to be reduced by 0.9% and for Model 2 the costs would need to be reduced by 18.4%.

**TABLE 5:** Potential cost savings of alternative models of care to the NHS

	No reduction in number of health care professionals seen after introducing model	Reduction by 50% in number of health care professionals seen	Reduction by 100% in number of health care professionals seen
Model 1	£1786.66	−£2490.34	−£6767.34
Model 2	£1573.67	−£2703.33	−£6980.33

## Discussion

This is the first paper to estimate the current costs of JoHD in the UK. A recent systematic review identified 19 articles, between 1980 and 2024, discussing economic costs of HD. That review highlighted the heterogeneity in methodology, geography, health care systems and lack of systematic disease stratification.^12^ Where reported, the mean age of study participants varied between 46.8 and 67.8 years. This heterogeneity is reflected in the overall costs in Europe of between $40,000 USD 2024–$125,020 USD 2024 (£251,572,020–£799,472,020 prs). None of the studies focused on JoHD and at least five excluded this category.

The nearest comparator to study is that of Jones et al^13^; they used the CSRI to collect resource use information in 131 UK patients in the European Huntington's Disease Network (EHDN) REGISTRY database.^32^ The mean age of the patients was 50 with a range of 18–78. It follows that a few of the patients in this study will have JoHD but the exact number is unknown. The sample was divided by the Shoulson and Fahn TFC rating scale^28^ (there were no patients in stage V) so an accurate comparison with the early and late stages above is not possible. The mean cost per year in 2013 was £21,605 (£23,892 inflated to 2020 prices), of which over 65% (£14,085 (£15,576 inflated to 2020 prices)) were due to informal care costs defined as personal care and help inside and outside the home based on an hourly rate of £242,013 (£26.49 inflated to 2020 prices). This is not a direct comparison with our costs to the family.

We have presented the first detailed summary of the costs of caring for a patient with JoHD. As may be expected, the costs increase with disease progression. A summary mean figure of £173,575 (95% CI: £118,350–£246,574) for all formal and informal care costs should be useful as a benchmark for assessing future changes to care.

Whilst it is not possible to predict that the results of current clinical trials will alter the natural history of the condition, it is important to have an assessment of the plans to manage children and young people in a specialist setting. The next step would be to develop a pilot project using one or both of our proposed MDT models. A limitation would be developing a suitable outcome measure. Quantitative outcome measures for children and young people with HD are, at best, limited so it is likely that a pilot project would require a qualitative assessment. A pilot project could also assess and potentially propose improvements to the transition arrangements between pediatric and adult care.

Given the rarity of JoHD and the naturally small sample size, dividing patients into mild, moderate and advanced stages meant that the number of participants in the middle stage was slightly lower in this study. A further limitation is that the categories were based on a clinical judgment rather than a validated clinical scale. Further replication in other contexts would be beneficial, as could the exploration of specific platforms for providing the virtual MDT (Model 2) particularly in a more current and technologically developed post‐covid era.

In conclusion, we have undertaken the first economic evaluation of the costs of JoHD in the UK. This important study documents the high costs associated with the care of people living with JoHD, particularly in the latter stages when the condition is also most harrowing. This should be borne in mind when supporting families. Our comparative modeling of two models of a systemic MDT approach (as compared with the current arrangement whereby MDTs from on ad hoc basis) shows the potential for a cost saving from each model assuming fewer local health care professionals are seen. These data have clear implications in the consideration of future JoHD models of care in an increasingly digital age.

## Author Roles

(1) Research project: A. Conception, B. Organization, C. Execution; (2) Statistical Analysis: A. Design, B. Execution, C. Review and Critique; (3) Manuscript Preparation: A. Writing of the first draft, B. Review and Critique.

T.A.Y.: 1A, 1B, 1C, 2A, 2B, 2C, 3B.

P.A.Y.: 1A, 1B, 1C, 2C, 3B.

A.H.: 1A, 1B, 1C, 2C, 3B.

J.T.: 2C, 2B.

H.S.: 1A, 1B, 1C, 2C, 3B.

O.W.Q.: 1A, 1B, 1C, 2C, 3B.

## Disclosures

**Ethical Compliance Statement:** The Study, Patient Information Sheets and Consent Forms were approved by the Yorkshire and Humber Leeds West Research Ethics Committee 14/YH/1032. Informed consent was written when interviews were face‐to‐face and verbally when via the telephone. We confirm that we have read the Journal's position on issues involved in ethical publication and affirm that that this work is consistent with those guidelines.

**Funding Sources and Conflict of Interest:** This research was funded by the National Institute for Health Research (NIHR) under its Research for Patient Benefit (RfPB) Programme (Grant Reference Number PB‐PG‐112‐29,056). This represents independent research funded by the National Institute for Health Research (NIHR) under its Research for Patient Benefit (RfPB) Programme (Grant Reference Number PB‐PG‐112‐29,056). The views expressed are those of the author(s) and not necessarily those of the NHS, the NIHR or the Department of Health. OQ was a Member of the Scientific Oversight Committee for the JOIN‐HD study for the Huntington's Disease Youth Organization until November 2024, unpaid. AH is a member of the Scientific Oversight Committee for the JOIN‐HD study, unpaid and member of the FELL‐HD steering committee, unpaid. HS is a member of the Scientific Oversight Committee for the JOIN‐HD study, unpaid and member of the FELL‐HD steering committee, unpaid.

**Financial Disclosures for Previous 12 Months:** OQ has written two medico‐legal reports on JoHD for West Yorkshire Police and Grimsby Crown Court and has royalties from Juvenile Huntington's and other trinucleotide repeat disorders OUP Oxford 2009. HS has royalties from Juvenile Huntington's and other trinucleotide repeat disorders OUP Oxford 2009.

## Supporting information


**Supporting Information S1.** is the response use questionnaire used to record data during the interviews with families.

**Supporting Information S2.** provides details of the unit costs and resource use frequencies for the 33 people with JoHD.

**Supporting Information S3.** details the estimated costs for the alternative multidisciplinary models of care: either a face‐to‐face MDT or a virtual MDT.

## Data Availability

The data that support the findings of this study are available from the corresponding author upon reasonable request.

## Footnotes

^1^
Room hire costs were obtained from two NHS and CCG websites in November 2020, these websites no longer provide this information but the sources are as follows: Sandwell and West Birmingham CCG: Optispace Clinical and Meeting Room Booking System, NHS Gateshead Health. Centralized Room Booking Policy.
