# Supplementary material for: Economic Cost of Current and Alternative Models of Multidisciplinary Care of Juvenile‐Onset Huntington's Disease
Source: Mov Disord Clin Pract. 2025 Nov 11;13(4):964–72. doi: 10.1002/mdc3.70433 (PMC13071353; doi:10.1002/mdc3.70433)
Supplement: Supplementary file 1 — Supporting Information S1. is the response use questionnaire used to record data during the interviews with families. [file MDC3-13-964-s003.docx]

Supplementary Materials 1: Resource Use Questionnaire


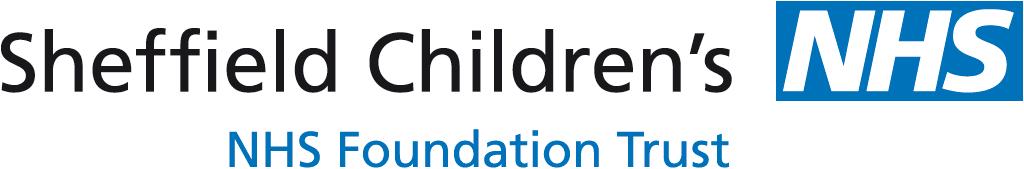
 [
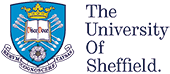
](http://www.google.co.uk/url?sa=i&rct=j&q=&esrc=s&source=images&cd=&cad=rja&uact=8&ved=0CAcQjRxqFQoTCOrig57S9sgCFcOrGgodfSIKUg&url=http://www.intohigher.com/uk/en-gb/our-centres/into-manchester/studying/ncuk-universities.aspx&psig=AFQjCNEZjOO1FhZ0lz1Y45Qxqh_IILGn6A&ust=1446722173779085)
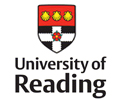


Thank you for your help with this telephone interview.

Our aim is to discover which services are being accessed by people/families with JHD as this will help us to both understand what care and support is already available and being accessed and to look at how this could be improved and enhanced. To be able to do this, we need to look at which services are currently provided.

**This is a comprehensive questionnaire and we do not expect you to complete it on your own** so we would like to contact you by telephone at a convenient time for you, to go through the questions and your answers and make any notes that would be relevant to our aim of improving the services offered to JHD patients and their families.

We would also like to ask you for the name and contact details of 3 professionals who you found to be most helpful. **Could you please think about the answer to this question in advance**? We would like to contact these people in the future with a view to discussing how care and support to JHD patients and their families could be provided more effectively and efficiently.

**If there is more than one person with JHD in the household, we will need to complete a questionnaire for each individual.**

**Section A**

**Background Details**

| 1 | Date of telephone Interview |  |
| --- | --- | --- |
| 2 | Name of Carer |  |
| 3 | Relationship to person with JHD |  |
| 4 | Name of person with JHD |  |
| 5 | DoB of person with JHD |  |
| 6 | Is there more than one person in the household with JHD | - Yes - No |

**Section B**

**We would now like to ask you for the name and contact details of 3 professionals who you found to be most helpful. We will discuss with them the care provision for JHD patients and families in general, so that we can look at how services can be improved and extended. No individual cases will be discussed.**

| Name | Profession | Contact details | How were they accessed? |
| --- | --- | --- | --- |
|  |  |  |  |
|  |  |  |  |
|  |  |  |  |

**Section C**

**This section is to give us basic data regarding the social, educational and employment background of the person with JHD.**

| 1 | Gender of person with JHD | - Male - Female | |
| --- | --- | --- | --- |
| 2 | Marital status of person with JHD | - Single - Partnership - Married | |
| 3 | Does the person with JHD have children? | - None - Yes Number: Age(s): | |
| 4 | Where is the person with JHD living? | - At home with family - Separate home outside of family without support - Separate home outside of family with support - Permanent residential care - Other (*Interviewer can make notes on the continuation sheet) | Age at commencement |
|  |  |  |  |
| 5 | Schooling or occupation | - Mainstream school - Specialist educational establishment (Please specify) - Home Schooled - College - University - Employment | Age at beginning and end |
|  |  |  |  |
| 6 | Is/was there an Education Health and Care Assessment (previously Statement of Special Educational and Disability Need)? | - Yes - No - Applied for but not granted | Age at which this was provided/ application made |

**Section D**

**In this section we would like to gain more information regarding the services accessed by the person with JHD.**

**In the last 12 months has the person with JHD had any of the following?**

|  |  | Was this due to JHD | Number of days | Notes |
| --- | --- | --- | --- | --- |
| 1** | Accident and Emergency | - Yes - No |  |  |
| 2** | In-patient admissions   - Mental health - Acute care - Intensive Care unit | - Yes - No - Yes - No - Yes - No |  |  |
| 3* | Day centre visits  (Number of days per week) | - Yes - No |  |  |
| 4** | Respite care | - Yes - No |  |  |
| 6** | Residential or Nursing home care | - Yes - No |  |  |
| 7** | Any other?  (*Interviewer can make notes on the continuation sheet) |  |  |  |

***Notes to interviewer**

*** Please enquire about the nature of the Day Centre**

**** Please check whether the appointments relate to JHD. If unsure, please make notes on the continuation sheet**

**Section E**

**In the last 12 months which professionals has the person with JHD seen?**

**(Notes to interviewer )**

***If one of the answers is Neuro-rehab, please specify by professional background if you can or ask if you can contact the team after the interview**

**** Please comment if the person with JHD is transitioning between paediatric and adult services**

|  | **Professional** | | | | **Number of contacts** | **Average duration of contact** | **Was this due to JHD** | |
| --- | --- | --- | --- | --- | --- | --- | --- | --- |
| 1 | GP | | | |  |  | - Yes - No | |
| 2 | Surgical outpatients | | - Adult - Paediatric - Not known | |  |  | - Yes - No | |
| 3 | Paediatrician (*Interviewer to check if this is a specialist or community paediatrician) | | | |  |  | - Yes - No | |
| 4 | Adult Medical Outpatients | | | |  |  | - Yes - No | |
| 5 | Neurologist | | | - Adult - Paediatric - Not known |  |  |  | |
| 6 | Psychiatrist | | | - Adult - Paediatric - Not known |  |  | - Yes - No | |
| 7 | Psychologist | | | - Adult - Paediatric - Not known |  |  | - Yes - No | |
| 8 | Clinical Psychologist | | | - Adult - Paediatric - Not known |  |  | - Yes - No | |
| 9 | Educational Psychologist | | | |  |  | - Yes - No | |
| 10 | Geneticist/Genetic counsellor | | | |  |  | - Yes - No | |
| 11 | Pain Clinic | | | |  |  | - Yes - No | |
| 12 | Counsellor | | | - Adult - Paediatric - Not known |  |  | - Yes - No | |
| 13 | Community Matron | | | - Adult - Paediatric - Not known |  |  | - Yes - No | |
|  | **Professional** | | | **Number of contacts** | **Average duration of contact** | **Was this due to JHD** | |  |
| 14 | Palliative care team | | | - Adult - Paediatric - Not known |  | - Yes - No | |  |
| 15 | District Nurse/Practice Nurse | | | - Adult - Paediatric - Not known |  | - Yes - No | |  |
| 16 | Mental Health Nurse | | | - Adult - Paediatric - Not known |  | - Yes - No | |  |
| 17 | Continence Nurse | | | - Adult - Paediatric - Not known |  | - Yes - No | |  |
| 18 | PEG Nurse | | | - Adult - Paediatric - Not known |  | - Yes - No | |  |
| 19 | Speech and Language Therapist | | | - Adult - Paediatric - Not known |  | - Yes - No | |  |
| 20 | Physiotherapist | | | - Adult - Paediatric - Not known |  | - Yes - No | |  |
| 22 | Occupational Therapist | | | - Adult - Paediatric - Not known |  | - Yes - No | |  |
| 23 | Dietician/Nutritionist | | | - Adult - Paediatric - Not known |  | - Yes - No | |  |
| 24 | Family Therapist | | | - Adult - Paediatric - Not known |  | - Yes - No | |  |
| 25 | Art Therapist | | | - Adult - Paediatric - Not known |  | - Yes - No | |  |
| 26 | Hydrotherapist | | | - Adult - Paediatric - Not known |  | - Yes - No | |  |
| 27 | Dentist | | | |  | - Yes - No | |  |
| 28 | Police | | | |  | - Yes - No | |  |
| 29 | Social Worker | | | |  | - Yes - No | |  |
| 29 | Paid carer/Personal Assistant | | | |  | - Yes - No | |  |
|  | **Professional** | **Number of contacts** | | | **Average duration of contact** | **Was this due to JHD** | |  |
| 30 | Acupuncturist | - NHS - Private | | |  | - Yes - No | |  |
| 31 | Homeopath | - NHS - Private | | |  | - Yes - No | |  |
| 32 | Herbalist | - NHS - Private | | |  | - Yes - No | |  |
| 33 | Aromatherapist | - NHS - Private | | |  | - Yes - No | |  |
| 34 | Reflexologist | - NHS - Private | | |  | - Yes - No | |  |
| 35 | In general, which form of transport do you use for appointments and how long does it take.  (*Interviewer can make notes on the continuation sheet) | | | | | | |  |
| 36 | Other services accessed  (*Interviewer can make notes on the continuation sheet) | | | | | | |  |

**Section F**

**Current medication of the person with JHD**

| Drug | Dosage |
| --- | --- |
|  |  |
|  |  |
|  |  |
|  |  |
|  |  |
|  |  |
|  |  |

**Section G**

**In the last 12 months has the person with JHD had any medical investigations?**

|  | Investigation |  | Reasons for test |
| --- | --- | --- | --- |
| 1 | MRI scans | - Yes - No |  |
| 2 | Genetic testing | - Yes - No |  |
| 3 | CT/CAT scan | - Yes - No |  |
| 4 | Electroencephalogram (EEG) | - Yes - No |  |
| 5 | Other  (*Interviewer can make notes on the continuation sheet) |  |  |

**Section H**

**Thinking about the current situation and not specifically the last 12 months. Does the person with JHD use any equipment?** **Could you tell us if this equipment was provided by the person with JHD or their family or was it provided by the NHS or Local Authority (LA) funding or from a charity?**

|  | **Aids or Devices** | **Approx when was equipment provided?** | **Age when provided** | **Who provided the equipment?** | **If applicable, who provided an upgrade?** |
| --- | --- | --- | --- | --- | --- |
| 1 | Wheel chair – self propelled |  |  | - Myself/family - NHS/LA - Charity | - Myself/family - NHS/LA - Charity |
| 2 | Wheelchair - Electric |  |  | - Myself/family - NHS/LA - Charity | - Myself/family - NHS/LA - Charity |
| 3 | Crutches or sticks |  |  | - Myself/family - NHS/LA - Charity | - Myself/family - NHS/LA - Charity |
| 4 | Walking frame |  |  | - Myself/family - NHS/LA - Charity | - Myself/family - NHS/LA - Charity |
| 5 | Commode |  |  | - Myself/family - NHS/LA - Charity | - Myself/family - NHS/LA - Charity |
| 6 | Bathing equipment |  |  | - Myself/family - NHS/LA - Charity | - Myself/family - NHS/LA - Charity |
| 7 | Toileting equipment |  |  | - Myself/family - NHS/LA - Charity | - Myself/family - NHS/LA - Charity |
| 8 | Pressure relieving cushions/mattress |  |  | - Myself/family - NHS/LA - Charity | - Myself/family - NHS/LA - Charity |
| 9 | Adapted eating utensils |  |  | - Myself/family - NHS/LA - Charity | - Myself/family - NHS/LA - Charity |
| 10 | Hoist |  |  | - Myself/family - NHS/LA - Charity | - Myself/family - NHS/LA - Charity |
| 11 | Medicalised bed |  |  | - Myself/family - Provided for me | - Myself/family - Provided for me |
| 12 | Any other aids or devices? (*Interviewer can make notes on the continuation sheet) |  |  | - Myself/family - NHS/LA - Charity | - Myself/family - NHS/LA - Charity |

**Section I**

**This section is to look at information regarding how the home has been adapted for the person with JHD or their family. Again, if any adaptations have been made we would like to know if the person with JHD has had to fund these themselves or whether the adaptations were funded by statutory services, Local Authority or a charity. If you are not sure who has provided the equipment then simply tell us that you don’t know. (*Note to interviewer – please record if the answer to the funding question is ‘Don’t Know’**

|  | **Adaptations to house** | **Approx how long ago was this done** | **Age when provided** | **Who provided the equipment?** | **If applicable, who provided an upgrade?** |
| --- | --- | --- | --- | --- | --- |
| 1 | Stairlift |  |  | - Myself/family - LA - Charity | - Myself/family - LA - Charity |
| 2 | Handrails |  |  | - Myself/family - LA - Charity | - Myself/family - LA - Charity |
| 3 | Ramps |  |  | - Myself/family - LA - Charity | - Myself/family - LA - Charity |
| 4 | Shower/bath alteration |  |  | - Myself/family - LA - Charity | - Myself/family - LA - Charity |
| 5 | Toilet alteration |  |  | - Myself/family - LA - Charity | - Myself/family - LA - Charity |
| 6 | Kitchen redesign |  |  | - Myself/family - LA - Charity | - Myself/family - LA - Charity |
| 7 | House extension |  |  | - Myself/family - LA - Charity | - Myself/family - LA - Charity |
| 8 | Door widening |  |  | - Myself/family - LA - Charity | - Myself/family - LA - Charity |
| 9 | Any other adaptations? (*Interviewer can make notes on the continuation sheet) |  |  | - Myself/family - LA - Charity | - Myself/family - LA - Charity |

**Section J**

**Thinking about daily activities and day to day living experience – what kind of support has been provided for the person with JHD in the last 12 months?**

|  |  |  | Average number of hours per week | Who provides this support |
| --- | --- | --- | --- | --- |
| 1 | Help with personal care (eg bathing/dressing) | - Yes - No |  | - Volunteer - Paid carer - Family member |
| 2 | Help inside the home (eg cooking/cleaning) | - Yes - No |  | - Volunteer - Paid carer - Family member |
| 3 | Help outside the home (eg shopping) | - Yes - No |  | - Volunteer - Paid carer - Family member |
| 4 | Help with accessing recreational activities | - Yes - No |  | - Volunteer - Paid carer - Family member |
| 5 | Any other help?  (*Interviewer can make notes on the continuation sheet) |  |  | - Volunteer - Paid carer - Family member |

**Section K**

**Thinking about any changes in employment or financial circumstances -** has caring for the person with JHD interfered with **the employment of you or the main carer** in the last 12 months?

***Note to interviewer – if the answer to Q1 is ‘No’ please go straight to Q5**

**- if the answer to Q1 is ‘Yes’ please complete Q2-4**

|  |  |  | Notes |
| --- | --- | --- | --- |
| 1 | Has affected employment/usual activities | - Yes - No |  |
| 2 | Days off work/usual activities | - Yes - No |  |
| 3 | Working part time due to caring for person with JHD | - Yes - No |  |
| 4 | Have had to give up work | - Yes - No |  |
| 5 | Not in paid employment | - Yes - No |  |
| 6 | Change of employment/occupation | - Yes - No |  |

**(*Interviewer can make further notes on the continuation sheet)**

**Has caring for the person with JHD affected the employment of the carer’s partner/rest of household/family unit?**

|  |  |  | Notes |
| --- | --- | --- | --- |
| 1 | Has had no effect | - Yes - No |  |
| 2 | Days off work | - Yes - No |  |
| 3 | Reduced working hours | - Yes - No |  |
| 4 | Had to give up work | - Yes - No |  |
| 5 | Change of employment/occupation | - Yes - No |  |

**(*Interviewer can make further notes on the continuation sheet)**

**Section L**

**In the last 12 months have you had to make phone calls or visits because of caring for someone with JHD? For example:**

**Can you estimate how much time you have spent making or changing appointments/ dealing with financial/personal issues for the person with JHD?**

**Can you estimate how much time you have spent making any visits not covered in the previous sections? This could be dealing with financial/personal issues for the person with JHD.**

**Thank you for your help in completing this questionnaire – your contribution is valued and will help towards our work in providing appropriate, effective and efficient models of care for persons with JHD**
