# Supplementary material for: Economic Cost of Current and Alternative Models of Multidisciplinary Care of Juvenile‐Onset Huntington's Disease
Source: Mov Disord Clin Pract. 2025 Nov 11;13(4):964–72. doi: 10.1002/mdc3.70433 (PMC13071353; doi:10.1002/mdc3.70433)
Supplement: Supplementary file 2 — Supporting Information S2. provides details of the unit costs and resource use frequencies for the 33 people with JoHD. [file MDC3-13-964-s001.docx]

## Supplementary Materials 2: Details of unit costs and resource use frequencies (N = 33)

|  | Numbers accessing | Mean and range | Unit cost (£) in 2020 | Unit Cost Source |
| --- | --- | --- | --- | --- |
| **Services accessed** | | | | |
| A&E | 16 (48.5%) | 0.94 (0 to 6) | £182 per visit | NHS National Cost Collection |
| Residential/nursing home care | 8 (24.2%) | 7 permanent residents  and 1 stay of 104 days | £4,971 (per week) | PSSRU |
| Respite care | 7 (21.2%) | 7.55 days (0 to 104) | £4700 (per day) | PSSRU |
| Day centre visits | 3 (9.1%) | 7.88 days (0 to 156) | £150 (per visit) | NHS National Cost Collection |
| In-patient (acute care) | 3 (9.1%) | 0.03 days (0 to 1) | £4,778 (per stay) | NHS National Cost Collection |
| In-patient (mental health) | 2 (6.1%) | 0.03 days (0 to 1) | £600 (per day) | NHS National Cost Collection |
| Other: prison | 1 (3.0%) | Throughout year of study | £42,670 (per prisoner per year) | UK Government |
| **Health Professionals Seen** | | | | |
| GP | 23 (69.7%) | 3.48 (0 to 12) | £39 (per visit)  £232 telephone – per hour | PSSRU |
| Speech and Language Therapist | 23 (69.7%) | 2.87 (0 to 12) | £112 (Child per visit)  £108 (Adult per visit) | NHS National Cost Collection |
| Neurologist | 20 (60.6%) | 1.27 (0 to 5) | £187 (per visit) | NHS National Cost Collection |
| Occupational therapist | 19 (57.6%) | 4.94 (0 to 52) | £155 (Child per visit)  £84 (Adult per visit) | NHS National Cost Collection |
| Dietician/nutritionist | 20 (60.6%) | 2.33 (0 to 12) | £92 (per visit) | NHS National Cost Collection |
| Dentist | 4 (12.1%) | 1.75 (0 to 24) | £62 (per visit) | PSSRU |
| Physiotherapist | 15 (45.5%) | 3.24 (0 to 52) | £110 (Child per visit)  £67 (Adult per visit) | NHS National Cost Collection |
| Social worker | 11 (33.3%) | 3.12 (0 to 52) | £52 (Child per hour)  £51 (Adult per hour) | PSSRU |
| District nurse/practice nurse | 10 (30.3%) | 8.6 (0 to 227) | £68 per hour | PSSRU |
| Paid carer/personal assistant | 10 (30.3%) | 6.56 (0 to 168) | £24 per hour | PSSRU |
| PEG Nurse | 10 (30.3%) | 1.18 (0 to 15) | £68 per hour | PSSRU |
| Continence nurse | 9 (27.3%) | 0.58 (0 to 6) | £90 per visit | NHS National Cost Collection |
| Psychiatrist | 8 (24.2%) | 1.94 (0 to 52) | £351 (Child per visit)  £259 (Adult per visit) | NHS National Cost Collection |
| Psychologist | 5 (15.2%) | 1.76 (0 to 52) | £330 (per visit) | NHS National Cost Collection |
| Clinical Psychologist | 6 (18.2%) | 3.73 (0 to 52) | £330 (per visit) | NHS National Cost Collection |
| Community matron | 6 (18.2%) | 2.5 (0 to 52) | £68 (per hour) | PSSRU |
| Surgical outpatients | 5 (15.2%) | 0.3 (0 to 3) | £148 (per visit) | NHS National Cost Collection |
| Paediatrician | 5 (15.2%) | 0.67 (0 to 16) | £296 (Neurology per visit)  £302 (Community per visit) | NHS National Cost Collection |
| Other | 5 (15.2%) | N/a | £102 (Optometry per visit)  £47 (Podiatrist per visit)  £135 (Family fun day)  £251 (Overnight palliative care)  £208 (Solicitor) | NHS National Cost Collection  NHS National Cost Collection  PSSRU  PSSRU  Civil Legal Aid |
| Educational psychologist | 4 (12.1%) | 0.18 (0 to 2) | £330 (per visit) | NHS National Cost Collection |
| Police | 3 (9.1%) | 1.27 (0 to 35) | £1 (per minute) | Home Office |
| Aromatherapist | 3 (9/1%) | 0.42 (0 to 4) | £53 (per session) | Find a therapy |
| Adult medical outpatients | 2 (6.1%) | 0.21 (0 to 3) | £148 (per visit) | NHS National Cost Collection |
| Mental health nurse | 4 (12.1%) | 0.54 (0 to 12) | £26 (per visit) | PSSRU |
| Art therapist | 3 (9.1%) | 3.52 (0 to 52) | £139 (Child per visit)  £139 (Adult per visit) | NHS National Cost Collection |
| Hydrotherapist | 1 (3.0%) | 6.31 (0 to 156) | £138 (per visit) | NHS National Cost Collection |
| Palliative care team | 2 (6.1%) | 2.30 (0 to 50) | £189 (per hour) | PSSRU |
| Reflexologist | 2 (6.1%) | 0.36 (0 to 6) | £39 (per session) | Median across pure reflexology & natural balance |
| Counsellor | 1 (3.0%) | 0.36 (0 to 12) | £21 (per hour) | Agenda for change |
| Family therapist | 1 (3.0%) | 0.79 (0 to 26) | £139 (per visit) | NHS National Cost Collection |
| Acupuncturist | 1 (3.0%) | 0.30 (0 to 1) | £55 (per session) | PROSPECTS |
| Herbalist | 1 (3.0%) | 0.30 (0 to 1) | $45 (per session) | Median across Lodge House, The Herbalist & Traditional herbalist |
| **Medications (See separate table below)** | | | | |
| **Investigations** | | | | |
| Genetic testing | 5 (15.2%) | N/a | £34 | NHS National Cost Collection |
| Electroencephalogram (EEG) | 4 (12.1%) | N/a | £39 | NHS National Cost Collection |
| Ultrasound | 2 (6.1%) | N/a | £37 | NHS National Cost Collection |
| X-ray | 1 (3.0%) | N/a | £29 | NHS National Cost Collection |
| Metabolic tests | 1 (3.0%) | N/a | £37 | NHS National Cost Collection |
| **Equipment used** | | | | |
| Bathing equipment | 20 (60.6%) | N/a | £673 | PSSRU |
| Wheelchair (self-propelled) | 16 (48.4%) | N/a | £458 (NHS)  £240 (Self) | NHS National Cost Collection  CareCo |
| Medicalised bed | 4 (12.1%) | N/a | £685 (NHS)  £1,088 (Self) | Care Co – basic type  Care Co |
| Hoist | 11 (33.3%) | N/a | £895 (NHS & Self) | Care Co |
| Pressure relieving cushions/mattress | 11 (33.3%) | N/a | £30 (NHS)  £36 (Self) | Care Co – basic type  Care Co |
| Adapted eating utensils | 3 (9.1%) | N/a | £7 (NHS & Self) | Care Co |
| Toileting equipment | 11 (33.3%) | N/a | £30 (NHS)  £45 (Self) | Care Co – basic type  Care Co |
| Commode | 6 (18.2%) | N/a | £50 (NHS)  £148 (Self) | Care Co – basic type  Care Co |
| Walking frame | 4 (12.1%) | N/a | £35 (NHS) | Care Co – basic type |
| **Adaptations** | | | | |
| Shower/bath | 15 (45.4%) | N/a | £673 | PSSRU |
| Handrails | 15 (45.4%) | N/a | £4.10 NHS  £7.92 (Self) | PSSRU  Screwfix |
| Ramps | 9 (27.3%) | N/a | £46 | PSSRU |
| Toilet alteration | 10 (30.3%) | N/a | £1,426 | PSSRU |
| House extension | 5 (15.2%) | N/a | £4,867 (NHS)  £4,751 (Self) | PSSRU  Designforme.com |
| Door widening | 5 (15.2%) | N/a | £77 | PSSRU |
| Stair lift | 4 (12.1%) | N/a | £279 (NHS)  £385.26 | PSSRU  Which |
| Garden access | 2 (6.1%) | N/a | £62 | PSSRU |
| Banister rails | 1 (3.0%) | N/a | £4.10 NHS  £7.92 (Self) | PSSRU  Screwfix |
| **Other costs** | | | | |
| Car journey | 18 (54.5%) | N/a | £0.45 per mile | Gov.co.uk |
| Taxi | 3 (9.1%) | N/a | £2.70 per mile plus £2.50 pick up charge | Taxi calculator.com |
| Bus | 2 (6.1%) | N/a | £2 per journey | First travel |
| Family carer/volunteer | 23 (69.7%) | N/a | £14.65 per hour | Office of National Statistics |
