# Supplementary material for: Economic Cost of Current and Alternative Models of Multidisciplinary Care of Juvenile‐Onset Huntington's Disease
Source: Mov Disord Clin Pract. 2025 Nov 11;13(4):964–72. doi: 10.1002/mdc3.70433 (PMC13071353; doi:10.1002/mdc3.70433)
Supplement: Supplementary file 3 — Supporting Information S3. details the estimated costs for the alternative multidisciplinary models of care: either a face‐to‐face MDT or a virtual MDT. [file MDC3-13-964-s002.docx]

## Supplementary Materials 3: Estimated costs of alternative multidisciplinary models of care

| Resource | Level | Source | Salary/hourly cost | Assumption model 1 | Cost model 1 | Assumptions model 2 | Cost model 2 |
| --- | --- | --- | --- | --- | --- | --- | --- |
| Keyworker | Band 7@60% | Agenda for change | £36,612 | 2 people | £43,934.40 | 2 people | £43,934.40 |
| Phone calls from keyworker |  | BT | 0.13 | 1 day worth of calls | £58.50 | 1 day worth of calls | £58.50 |
| Meeting room hire |  | Level 1  Level 2 | £60  £96 | 6 meetings per year | £360.00  £576.00 | 6 meetings per year | £360.00  £576.00 |
| IT support | Band 5 | Agenda for change | £12.55 (hour) | N/a | N/a | ½ day per month | £602.40 |
| Zoom licence cost |  | Zoom us | £11.99 per month | N/a | N/a |  | £143.88 |
| **MDT regional team** | | | | | | | |
| Neurologist | Consultant | British medical association | £83,972 | 6 days per year plus travel time | £3,875.63 | 6 days per year | £1,937.82 |
| Speech & Language | Band 7 | Agenda for change | £18.72 | 6 days per year plus travel time | £1.684.80 | 6 days per year | £842.40 |
| Occupational therapy | Band 6 | Agenda for change | £15.68 | 6 days per year plus travel time | £1,411.20 | 6 days per year | £705.60 |
| Palliative care | Band 7 | Agenda for change | £18.72 | 6 days per year plus travel time | £1.684.80 | 6 days per year | £842.40 |
| Psychiatrist | Consultant | British medical association | £83,972 | 6 days per year plus travel time | £3,875.63 | 6 days per year | £1,937.82 |
| Social worker | Band 6 | Agenda for change | £15.68 | 6 days per year plus travel time | £1,411.20 | 6 days per year | £705.60 |
| **Local team** | | | | | | | |
| Consultant | Consultant | British medical association | £83,972 | 3 hourly contacts per year | £121.11 | 3 hourly contacts per year | £121.11 |
| Occupational/physio therapy | Band 6 | Agenda for change | £15.68 | 3 hourly contacts per year | £47.04 | 3 hourly contacts per year | £47.04 |
| Social worker | Band 6 | Agenda for change | £15.68 | 3 hourly contacts per year | £47.04 | 3 hourly contacts per year | £47.04 |
| **Family** | | | | | | | |
| Family travel |  | AA | £0.52 per mile | 100 miles (car) | £3,779.28 | 10 miles (car) | £377.93 |
| Family time lost to usual activities |  | Office of national statistics | £13.75 | Assume each meeting takes 1 day | £7,425 | Average hourly wage | £3,960 |
